# Mapping Grain Iron and Zinc Content Quantitative Trait Loci in an Iniadi-Derived Immortal Population of Pearl Millet

**DOI:** 10.3390/genes9050248

**Published:** 2018-05-11

**Authors:** Sushil Kumar, Charles Tom Hash, Thirunavukkarasu Nepolean, Mahesh D. Mahendrakar, Chellapilla Tara Satyavathi, Govind Singh, Abhishek Rathore, Rattan S. Yadav, Rajeev Gupta, Rakesh K. Srivastava

**Affiliations:** 1 Plant Biotechnology Centre, SK Rajasthan Agricultural University, Bikaner 334006, India; sushil254386@yahoo.com (S.K.); govindsingh10@rediffmail.com (G.S.); 2 International Crops Research Institute for the Semi-Arid Tropics (ICRISAT), Patancheru, Telangana 502324, India; mahendrakar.mahesh@gmail.com (M.D.M.); a.rathore@cgiar.org (A.R.); g.rajeev@cgiar.org (R.G.); 3 Centre of Excellence in Biotechnology, Anand Agricultural University, Anand, Gujarat 388110, India; 4 International Crops Research Institute for the Semi-Arid Tropics (ICRISAT), Niamey 8001, Niger; ct_hash@yahoo.com; 5 Indian Agricultural Research Institute (IARI), New Delhi 110012, India; tnepolean@gmail.com (T.N.); c.satyavathi@gmail.com (C.T.S.); 6 Institute of Biological, Environmental and Rural Sciences, Aberystwyth University, Aberystwyth SY23, 3EB, UK;rsy@aber.ac.uk

**Keywords:** pearl millet, QTL mapping, iron and zinc content, DArT, SSR, RILs, QTL

## Abstract

Pearl millet is a climate-resilient nutritious crop requiring low inputs and is capable of giving economic returns in marginal agro-ecologies. In this study, we report large-effect iron (Fe) and zinc (Zn) content quantitative trait loci **(**QTLs) using diversity array technology (DArT) and simple sequence repeats (SSRs) markers to generate a genetic linkage map using 317 recombinant inbred line (RIL) population derived from the (ICMS 8511-S1-17-2-1-1-B-P03 × AIMP 92901-S1-183-2-2-B-08) cross. The base map [seven linkage groups (LGs)] of 196 loci was 964.2 cM in length (Haldane). AIMP 92901-S1-183-2-2-B-08 is an *Iniadi* line with high grain Fe and Zn, tracing its origin to the Togolese Republic, West Africa. The content of grain Fe in the RIL population ranged between 20 and 131 ppm (parts per million), and that of Zn from 18 to 110 ppm. QTL analysis revealed a large number of QTLs for high grain iron (Fe) and zinc (Zn) content. A total of 19 QTLs for Fe and Zn were detected, of which 11 were for Fe and eight were for Zn. The portion of the observed phenotypic variance explained by different QTLs for grain Fe and Zn content varied from 9.0 to 31.9% (cumulative 74%) and from 9.4 to 30.4% (cumulative 65%), respectively. Three large-effect QTLs for both minerals were co-mapped in this population, one on LG1 and two on LG7. The favorable QTL alleles of both mineral micronutrients were contributed by the male parent (AIMP 92901-deriv-08). Three putative epistasis interactions were observed for Fe content, while a single digenic interaction was found for Zn content. The reported QTLs may be useful in marker-assisted selection (MAS) programs, in genomic selection (GS) breeding pipelines for seed and restorer parents, and in population improvement programs for pearl millet.

## 1. Introduction

Pearl millet [*Pennisetum glaucum* (L). R. Br.] is a climate-resilient crop of the semi-arid tropics of Asia and Africa [1]. It is a major source of energy, proteins, vitamins, and minerals for millions of the poorest people in these regions [2]. Drought and salinity tolerance and high water-use efficiency of pearl millet place it as one of the most preferred cops in the advent of changing climate characterized by global warming, mounting temperatures, growing water shortages, and escalating salinity. Globally, pearl millet is a leading crop for subsistence- and market-oriented crop-livestock production systems in the harsher environments of Asia and Africa. Globally, pearl millet is a staple food for above 90 million people [3]. 

For pearl millet, several molecular marker systems, such as restriction fragment length polymorphisms (RFLPs) [4], sequence tagged sites (STSs) [5], amplified fragment length polymorphisms (AFLPs) [6], single-stranded conformational polymorphism (SSCP) [7], gSSRs (simple sequence repeats) [8,9,10], EST-SSRs (expressed sequence tag- simple sequence repeats) [11,12,13,14,15], and diversity array technology (DArT) [14,15] have been deployed in last 25 years. Among the different marker systems, SSRs is considered a powerful system for marker-assisted breeding (MAB) because of its co-dominant nature, abundance, reproducibility, and variability. However, the relatively high cost per data point and low genome coverage restrict the application of SSRs in quantitative trait loci (QTL) mapping and marker-assisted selection programs. The current trend in cereal molecular breeding is to deploy high-throughput and cost-efficient markers, such as single nucleotide polymorphism (SNP) [16] and DArT [17]. DArT, a sequence-independent alternative to the common gel-based marker system, can overcome the limitations of the present marker systems in pearl millet. DArT is developed using the metagenome, offers a high multiplexing level, and can even include diversity surveys of newly developed varieties for analysis [18], as the same platform is used for discovering and scoring polymorphic markers. DArT has been employed for genetic mapping, genotyping, and diversity assessment in cereals and millets [15,19,20]. Recently, DArT-based linkage maps have been developed in pearl millet [14,15].

During QTL mapping, the type and size of the mapping population determine the gene effect for a trait [21,22,23]. Among various types of mapping population, recombinant inbred lines (RILs) population constitutes a permanent resource, as it can be replicated indefinitely. Moreover, because of several rounds of meiosis and recombination cycles, mapping resolution in RILs is more than in F_2_ and doubled haploid (DH) populations. Therefore, RIL populations can determine the map positions of even tightly linked markers. However, the mapping resolution and the ability to determine the marker order is fundamentally dependent on the population size [24]. The available literature on pearl millet (Table S1) displays that genetic maps are constructed using F_2_/RIL with a population size of <200. Hence, the objective of this study was to construct a linkage map using DArT markers in a large-sized RIL mapping population of pearl millet. 

Deficiencies or insufficient intakes of nutrients, especially vitamins or minerals, lead to several dysfunctions and diseases in humans that together are referred to as micronutrient malnutrition or hidden hunger. More than three billion people in the world today may be affected by micronutrient malnutrition [25,26,27]. Carbohydrate-rich, cereal-based monotonous consumption of foods with low contents and reduced bioavailability of Fe and Zn has been considered a major reason for Zn and Fe deficiency, especially among resource-poor women, infants, and children in the developing countries [28,29]. Targeted strategies available to alleviate micronutrient deficiencies include dietary diversification, food fortification, and supplementation. The breeding of agricultural crops for higher nutrient levels (or ‘biofortification’) is an emerging approach that complements the existing “toolbox” of interventions. 

Being a multipurpose crop, pearl millet is an important food for the undernourished people of the semi-arid tropics. Moreover, it is also well established that high metabolizable energy, proteins, and micronutrients make pearl millet an extremely nutrient-rich cereal [14]. Hence, pearl millet has been targeted for breeding-based biofortification to combat sinister forms of micronutrient malnutrition and hidden hunger. Its micronutrients contribution, particularly of iron (Fe) and zinc (Zn), varies from 30 to 50% of the intake of both micronutrients from cereals [14]. Therefore, the newly developed mapping population was utilized to map the QTLs for grain Fe and Zn content in an *Iniadi*-genetic background, known for high grain Fe and Zn content.

## 2. Materials and Methods

### 2.1. Plant Material

A segregating population of 317 F_6_-RILs was obtained from the cross of two inbred lines (ICMS 8511-S1-17-2-1-1-B-P03 × AIMP 92901-S1-183-2-2-B-08). The inbred ICMS 8511-S1-17-2-1-1-B-P03 (hereafter, ICMS) was derived from an experimental synthetic variety of pearl millet, namely, ICMS 8511, and bred at International Crops Research Institute for the Semi-Arid Tropics (ICRISAT)-Patancheru by random-mating a small number of inbred lines. It has small grain size and low grain Fe and Zn content [30]. The second inbred AIMP 92901-S1-183-2-2-B-08 (hereafter AIMP) was also developed at ICRISAT-Patancheru by selfing and selection within the released improved open-pollinated variety AIMP 92901 (Samrudhi-MP-282), which was bred collaboratively by ICRISAT and the pearl millet breeding team at National Agricultural Research Project (NARP) Aurangabad, Maharashtra, using the C4 cycle bulk of ICRISAT’s Bold Seeded Early Composite (BSEC) as a base population. AIMP 92901-S1-183-2-2-B-08 is an *Iniadi* parent tracing its origin to the Togolese Republic, West Africa. The lines originating from this region are known to have a high grain Fe and Zn content. This fertilizer-responsive, short-statured, and downy mildew-resistant inbred produces large grain having high grain Fe and Zn content [30]. Thus, the population segregated for grain iron and zinc content along with many other agronomic traits.

A single F_1_ plant produced after the initial plant × plant cross of both parents was selfed to produce F_2_ seeds, and the progeny was then advanced through single-seed descent (SSD) till the F_6_ generation, after which it was maintained as self-bulk. During generation advance, hand-sowing was done for each entry to provide five plants in every plot of 1 m length for producing self-seeds. Bulk seeds were harvested from the middle three out of five plants in each plot. For each entry, one selfed panicle was also harvested to provide a stock of nucleus seeds to maintain the purity and for future use in the advancing of every individual RIL.

### 2.2. DNA Extraction and Marker Analysis

Genomic DNA was extracted from the 15-days-old leaves of the population using sodium dodecyl sulphate (SDS) buffer as mentioned in Kumar et al. [14]. DNA quality was assessed by electrophoresis on agarose gels, and the DNA concentration was adjusted to 10 ng μL^−1^. Though the cost per data point of SSR is higher than that of DArT, on the basis of previous SSR based maps, SSRs are helpful in the designation of linkage groups and in the anchoring of the DArT markers in the new mapping population. Therefore, a total of 342 fluorescently labeled SSRs primer pairs of the *Xpsmp* series [8,9,31], *Xctm* series [10], *Xicmp* series [12], and *Xipes* series [13] were used for a polymorphism survey of the parental lines. Polymerase chain reaction (PCR) amplification of the *Xpsmp*, *Xctm*, and *Xicmp* series primers was performed in a reaction mixture (5 µL) containing 1 µL (10 ng) of genomic DNA, 0.5 µL of each primer (2 pmole/µL), 0.5 µL of 10× PCR buffer (ABI), 0.2 µL of 25 mM MgCl_2_, 0.2 U Taq DNA polymerase (Applied Biosystems, Foster City, CA, USA), 0.5 µL of 2 mM dNTPs, and double-distilled sterilized water. The selective amplification was carried out using a touchdown PCR method consisting of one cycle at 94 °C for 5 min, followed by five cycles over which the annealing temperature was decreased by 1 °C per cycle, and a final step of 30 cycles at 94 °C for 30 s, 52 °C for 30 s, and 72 °C for 1 min. M13-tailed *Xipes* series primers were amplified as per Rajaram et al. [13].

After checking the amplicons in 1.2% agarose gel, the SSR amplicons were resolved on capillary electrophoresis (3730 DNA fragment analyser, Applied Biosystems, USA). The data were analyzed using GeneMapper software (Applied Biosystems, USA) for estimating the sizes of the PCR amplicons by comparison with the internal size standard LIZ^500^ (Applied Biosystems). A DNA marker was considered to be valid if it had a peak height of at least 100 fluorescent units.

For DArT analysis, DNA samples at 50 ng μL^−1^ concentration were submitted to the Genotyping Service Laboratory (GSL) at ICRISAT-Patancheru. A set of 6912 DArT clones developed by *Pst*I/*Bam*II complexity reduction at ICRISAT was used for population genotyping. DNA from RILs and both parental lines were genotyped with DArT, using a previously established protocol [15].

### 2.3. Construction of Linkage Map

To analyze the segregation pattern of markers for assessing deviations from the expected Mendelian ratio (1:1), a χ^2^-test was performed. Initially, markers with the goodness of fit were chosen for map construction, but later some of the distorted markers were also integrated into the map on the logarithm of the odds (LOD) value >3.0. The polymorphic markers were grouped into different linkage groups, using LOD scores ranging from 3 to 16 by Mapmaker/Exp 3.0 [32]. Markers within the groups were ordered by the “RIPPLE”, “BUILD”, and “TRY” commands in Mapmaker/ExP. The inter-marker distance was calculated using the Haldane mapping function (cM), and the maps were visualized using MapChart 2.2 [33].

### 2.4. Experimental Conditions and Phenotyping of the Population

The field trial was performed in an α-lattice design on Alfisol fields at ICRISAT Patancheru with two replications in one environment during summer (2010). The trial was carried out with a total of 324 entries (317 RILs + repeated parental line) in 18 blocks of 18 plots. Each line was sown in two-row plots (length 4 m) with 60 cm inter- and 15 cm intra-row spacing to produce bulks of selfed grains for iron and zinc analysis. 

Threshed seeds were cleaned and oven-dried at 80 °C for 2 h. The dried seeds were ground with a cyclone sample mill to get sample powder (Udy Corporation, Fort Collins, CO, USA). One gram of grain powder was digested in a triacid mixture of 5:2:1 by volume of concentrated nitric acid, sulfuric acid, and perchloric acid using an electric hot plate until smoke appeared. The minerals analysis (Fe at 248.3 nm wavelength and Zn at 213.9 nm wavelength) of the seeds was conducted in an Atomic Absorption Spectrophotometer (Model: Spectra AA 20, Varian, Palo Alto, CA, USA), according to Velu et al. [30]. For the preparation of the standard solution from the stock solution of 10 mg L^−1^ Fe or Zn, aliquots of 1, 2, 3, 4, and 5 mL were pipetted out into well-labeled 25 mL volumetric flasks. For each flask, the volume was made up to 25 mL with 0.5% H_2_SO_4_. These sets of standards were used for the calculation of the instrumental calibration curve for both minerals. For error control, the samples were measured in sets of 40 samples containing three blanks and two standard samples with one check sample of known mineral elements content. The grain Fe and Zn content were computed as parts per million (ppm).

### 2.5. Statistical Analysis

The phenotypic variance was partitioned using the residual maximum likelihood (ReML) algorithm with a mixed model, where replication and block were considered to be fixed effects, while genotypes were random effects, to obtain the best linear unbiased predictions (BLUPs) of the performance of genotypes for each observed trait. Pearson correlation coefficients were determined for the studied traits, namely, Fe and Zn content (ppm) using ‘PROC CORR’ in SAS (SAS Institute Inc., Cary, North Carolina, USA). Because a normal distribution could not be assumed for all the observed variables, Spearman’s rank correlation (rs) was also used as a robust estimation of the correlation coefficient. Similarly, genotypic correlations were also estimated. The significance of the correlation coefficients at *p* ≤ 0.05 and 0.01 was indicated as * and **, respectively. Broad-sense heritability on a plot means basis was computed from the estimates of genetic (σ^2^g) and residual (σ^2^e) variances using the progeny means across RILs in each environment for both traits, using PROC MIXED in SAS. Heritability (H^2^) was estimated as explained by Falconer [34].

### 2.6. Quantitative Trait Loci Detection

Composite interval mapping (CIM) [35] with best linear unbiased prediction (BLUP) data of each trait was used to identify QTL using PLABQTL [36] with two centiMorgan (cM) increments. To declare a putative QTL as statistically significant, a minimum LOD score of three was fixed according to the Bonferroni correction. The critical LOD threshold was analyzed empirically for each trait using 1000 permutation runs. The proportion of the phenotypic variance explained by the QTL was determined by the estimator R^2^ (%). The putative QTLs detected for each of the respective traits were assigned to linkage groups on the basis of the map positions of their flanking markers. 

## 3. Results and Discussion

### 3.1. Linkage Map

Linkage maps are important pre-requisite for QTL mapping and for marker-assisted breeding programs. RIL is the most favored mapping population, but the accuracy of the mapping resolution depends on the population size. So far, in pearl millet, linkage maps have been predominantly constructed using F_2_ populations and, up to certain extent, RILs. The number of genotypes in individual populations of pearl millet was less than 190 individuals, which is lower than the theoretically required size to achieve precise mapping [37]. The RIL population, namely, 81B × ICMP 451, presently accommodating 321 loci (258 DArTs and 63 SSRs) was developed from only 140 RILs [15]. However, the here presented mapping population with 317 RILs is the largest mapping population in pearl millet.

During the polymorphism survey, a set of 235 DArT clones (3.3%) was found polymorphic between both parents. The number of polymorphic DArT clones was comparatively lower than that found by Supriya et al. [15]. In the case of SSRs, 112 (33%) SSRs out of 342 were polymorphic and comprised 18% of *Xipes*, 13% of *Xpsmp*, and 2% of *Xicmp* markers. To construct the framework linkage map, a small set of 33 polymorphic SSRs was used for genotyping the RILs. The SSR polymorphism level was lower than that determined by Senthilvel et al. [12], which was 74%. The reason for a low polymorphism in the present study may be the closer relatedness of the mapping population parents in the present study compared to that of Senthilvel et al. [12]. 

**Figure 1:**
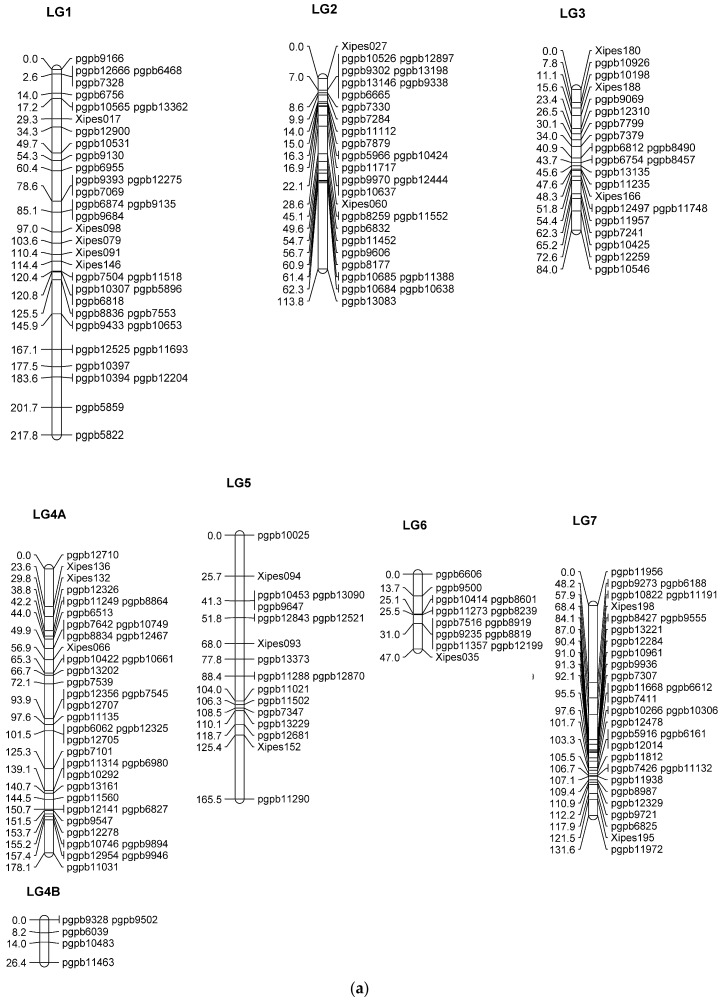

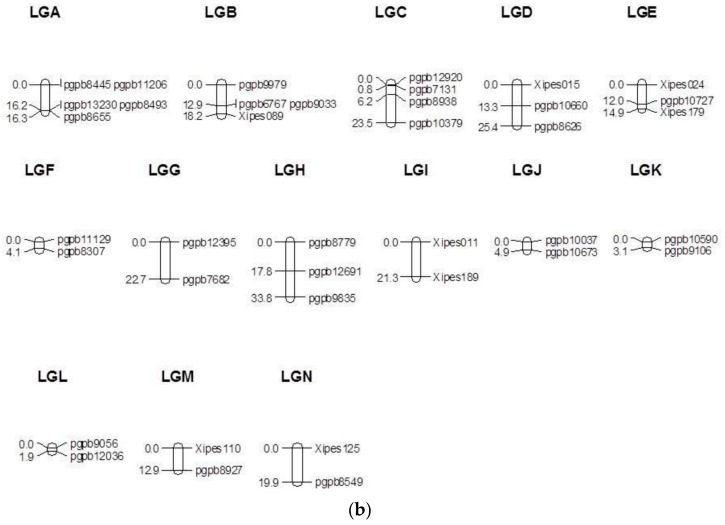
(**a**) Base linkage map (linkage groups LG1–LG7) of the pearl millet recombinant inbred line (RIL) population based on the cross (ICMS 8511-deriv × AIMP 92901-deriv-08); (**b**) Linked fragments from the genetic map of the pearl millet RIL population based on the cross (ICMS 8511-deriv × AIMP 92901-deriv-08).

As far as the positioning of the SSR markers on the genetic map, all SSRs mapped on the same linkage group (LG) as in previous pearl millet maps reported by various researchers [12,14,15,38]. A set of 75% of DArT loci was polymorphic, which is similar to the result of Kumar et al. [14] but is lower than the percentage found by Supriya et al. [15], who reported 80% polymorphism. Out of 235 polymorphic markers, 177 DArTs and 19 SSRs were assigned to seven previously established pearl millet linkage groups (base map), (Figure 1a), in which LG4 had two sub-groups (LG4A and LG4B). Thirty-four loci (28 DArT and 6 SSRs) not linked to any of the existing LGs and were grouped in 14 small groups (LG A to LG N), (Figure 1b), with markers ranging from two to five per group. Similar to the current study, Kumar et al. [14] also reported small LGs in the pearl millet linkage map. Moreover, attempts for linking any such small segments to any major group drastically increased the length of that major group, with huge inter-marker distances. Hence, these 14 segments were kept separate and treated as small LGs. The 34 unmapped markers either had a too high degree of segregation distortion or were positioned too distantly in relation to the next markers assigned to the linkage groups and were presumably on the distal ends of the LGs. The un-mapping of polymorphic markers in pearl millet was also reported earlier [14,15] 

In the current study, the distribution of DArT throughout the chromosome was uniform. The map of seven LGs consisting of 196 loci was 964 cM in length, as shown in Table 1. The average length of the LGs in this base map was 121 cM, and the average number of loci per LG was 24.5. LG1 was the longest, having 38 loci with a distance of 218 cM. The shortest LG in pearl millet on the basis of the previous studies of Gulia [39] and Yadav et al. [38] with four (30.2 cM) and nine markers (15.4 cM), respectively, was LG3; however, in the present study, LG6 was found to be the shortest LG (47 cM), holding 13 markers only. This indicates that the marker number was not sufficient in the previous investigations [14,15] to cover the full length of LG3. The length of LG2 was one-third of that previously reported in the DArT map of pearl millet.

The present map had a length greater than the one reported by Yadav et al. [38] and Gulia [39], and smaller than in the other maps [14,15]. The map covers 964 cM, which is comparable to the 815 cM of the map recently developed by Sehgal et al. [40], and 84% of the intervals were less than 10 cM, which is comparable with the intervals reported by Supriya et al. [15].

The average inter-marker lengths on the base map ranged from 3.6 cM (LG6) to 9.2 cM (LG5). Among the small segments, LG H was the longest (34 cM), while LG I was the shortest (1.9 cM). The average distance between markers in the base map was a little higher in the DArT linkage map of Supriya et al. [15]. DArT markers in large-sized population constitute a significant improvement in marker density and, possibly, inter-marker distance. A positive correlation was found between the number of mapped markers and the length of linkage groups. For instance, LG1 spans a distance of 219 cM with 33 markers, and LG4B, with 5 loci, spans a distance of 26 cM. Similar observations were recorded in an earlier DArT-based map of pearl millet study [15]. The markers that were unlinked to any of the major LGs will be merged into larger LGs with the availability of an additional set of polymorphic markers in the near future. 

The improved inter-marker distance of the current map, as compared to previously reported pearl millet maps [14,15,38,39,41], developed on a variable size population and a different number of markers, indicates that this map is one of the densest genetic maps for pearl millet. 

### 3.2. Segregation Distortion and Inter-Marker Gaps

RILs usually show segregation distortion, because, during the RIL development process, many recessive lethal genes become homozygous, and their expression caused failure to contribute seeds to subsequent generations, consequently resulting in a skewed population [14]. It was found that 60% of loci showed segregation distortion in the present linkage map. Distorted markers showing *p* > 0.001 were also used for mapping in the framework linkage groups. Segregation distortion was found in both maternal and parental loci, although it was much higher in ‘ICMS’, the female parent of our mapping population. In previous studies, segregation distortion in favor of the female parent alleles was observed [15,42]. Out of 196 markers on the base map, the segregations of 118 DArTs and 14 SSRs were distorted, and, of these, 60 were skewed towards AIMP, and these loci were distributed across all seven LGs. In pearl millet, pollen abortion is more common than abnormalities in the female gametes, leading to a relatively greater loss of male parent alleles and the ensuing skewness towards the female parent. The distorted markers mapped on LG1 and LG2 were skewed towards the alleles of the male parent (AIMP), while most of the distorted markers were skewed towards the alleles of the female parent (ICMS) in the remaining LGs. Most of the loci on linked fragments were skewed towards ICMS.

Distortion from expected Mendelian segregation has been observed previously in barley [43,44], rice [45,46], maize [47,48], wheat [49,50,51], and pearl millet [15,39]. It has been suggested that the protogynous nature of pearl millet also contributes to segregation distortion [4]. Residual heterozygosity and inbreeding depression during inbred development may also contribute, as previously demonstrated by Cloutier et al. [52] and Livingstone et al. [53]. At the same time, the residual heterozygosity existing in some RIL may be beneficial, since deleterious genetic combinations in the form of lethality or reduced fitness can be overcome. In contrast to previous studies, the segregation of almost all loci of LG3 was distorted, which may explain part of the increase in LG3 map length.

The characteristic feature of pearl millet linkage maps is the presence of large gaps between centromere and telomere. LG4 was split into two pieces, suggesting additional polymorphic markers are needed to fill-up the gap between these two pieces. Senthilvel et al. [12] also reported a similar big gap in LG4. The previously constructed framework map for the cross ICMB 841-P3 × 863B-P2 had large gaps in LG2 and LG7 [12,54]. This new population also showed a large gap (>25 cM) in LG2, LG5, and LG7, for which the most probable reason could be the extreme localization of recombination at the ends of LG2 and LG7 [38]. According to various researchers, large gaps in the distal regions reflect regions of high recombination, rather than a lack of markers in these regions [12,41,55].

It is, however, possible, on the other hand, that these linkage groups are still incomplete, and genomic resources can be extended to develop new markers that are located on the distal regions of the linkage groups. Moreover, areas of low marker density and gaps may correspond to regions of similar ancestry or identity by descent in the germplasm included in the initial diversity representation for the development of the DArT markers [56]. In the case of potato, a gap was observed, and the authors postulated that this could be due either to recombination hot spots or to fixation (homozygosity) of the potato genome [57]. The number of large gaps has decreased in the present study compared to previous linkage maps, although there is still the possibility to map more markers to saturate these gaps.

### 3.3. Variance Components and Heritability 

There is a great variation of mineral and agronomic traits in pearl millet. The parents of this RIL population showed statistically significant divergent phenotypes for both the traits studied. The differences between BLUP means of RILs and AIMP 92901-deriv-08 were significant for both traits. A very wide range of variation for both traits was detected among the RILs of this mapping population (Table 2). The genotypic components of variance (σ^2^g) for both traits were significant in this RIL population, and the operational heritability (H^2^) estimates were very high, ranging from 0.83 (Zn) to 0.88 (Fe) (Table 3). The relatively high operational heritability indicates that much of the phenotypic variance in the RIL populations is genetically controlled, and QTLs can be mapped with a reasonable degree of reliability, given an appropriate genetic map of this RIL population. Broad-sense operational heritability was generally high enough to permit effective QTL mapping, indicating moderately high to high proportions of genetic variance for the traits studied.

### 3.4. Correlation Analysis

The knowledge of the associations between nutritional and agronomic traits will enable the breeders to decide suitable selection/breeding program criteria for the simultaneous genetic improvement of complex and associated traits like mineral content. The correlation between grain Fe and Zn contents has been studied in several crops, with the results, by a large, showing similar trends. Both genotypic (9.4**) and phenotypic (9.3**) associations between Fe and Zn were very strong and significantly positive. This may point to common molecular mechanisms controlling the uptake and metabolism of these minerals in grains or to common transporters controlling the movement of these minerals within the plants [58,59]. The co-segregation of genes for these traits might be the reason of the strong association between these minerals in both populations. The direction and intensity of the association suggest that there are good opportunities for the simultaneous genetic improvement of both micronutrients by co-transferring superior alleles controlling these traits into the genetic backgrounds of elite lines [30]. Genotypic correlations were observed to be higher than their corresponding phenotypic correlations for all the traits, suggesting less interaction between the genetic makeup of the traits and the environmental conditions. The higher genotypic correlation coefficients compared to the phenotypic coefficients also are an indicator that there are inherent relationships between the traits studied. This finding is in agreement with those of Khairwal et al. [60] and Ezeaku and Mohammed [61].

### 3.5. Frequency Distributions

**Figure 2:**
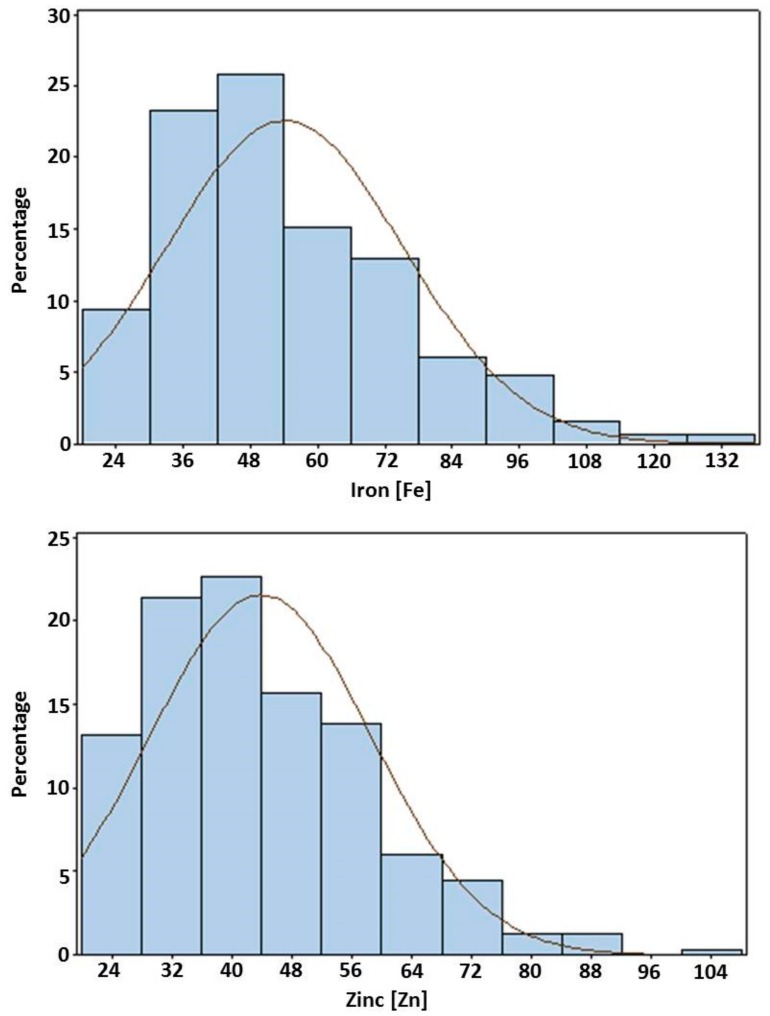
Frequency distribution of grain iron and zinc best linear unbiased predictions (BLUPs) among RILs of the ICMS 8511B (P1) × AIMP 92901-deriv-08 (P2)-based mapping population grown at ICRISAT-Patancheru.

The frequency distributions for the studied traits are given in Figure 2. The histogram of the RILs represents the frequency distributions of the overall means for both traits in the genotypes. In the case of the ICMS 8511-deriv × AIMP 92901-deriv-08 population, the distributions of RILs for Fe and Zn was skewed toward the ICMS 8511-deriv values. The bimodal distribution indicates the presence of one major genomic segment influencing the trait, with modifiers contributing to its quantitative variation. For both traits, the phenotypic normal distributions and transgressive segregations in the RIL population indicate polygenic inheritance. The presence of asymmetry in the distribution or skewness in the plot with transgressive segregation is indicative of epistatic interactions [62,63]. The presence of transgressive segregation also indicates the occurrence of genetic recombination [34], which points out that both favorable and unfavorable alleles for the trait studied are scattered between the parents.

### 3.6. Quantitative Trait Loci for Grain Iron (Fe) and Zinc (Zn) Content and Epistasis

The base map accommodated eight putative QTLs out of 11 detected for Fe content of self-pollinated grain in the summer 2010 dataset. R^2^ values (phenotypic expression) for individual QTLs ranged from 9 to 31.9%, while the R^2^ for their final simultaneous fit was 74.6%. The range of R^2^ was higher than that found by Kumar et al. (2016). A major putative QTL with R^2^ of 31.9% and a LOD value of 25.36 was detected on LG1. It had an additive effect of 9.7 ppm, and the favorable allele was the from high-Fe parent AIMP 92901-deriv-08 (Table 4). Compared to the present study, Kumar et al. [14] reported a major QTL for Fe on LG3. This indicated the role of the genome content in the parental genotypes. The result of one major QTL on LG1 and numerous putative QTLs with small effect is indicative of the probable complexity of inheritance of this trait, with effects distributed across the whole genome contributing to the control of uptake, accumulation, and content of this mineral micronutrient. The existence of multiple QTLs for grain iron content was also identified in different crops, like rice [64] and *Medicago* [65]. On the basis of the single-environment data analysis, three putative QTL × QTL interactions (QQI) were observed for Fe. 

Eight putative QTLs for selfed grain Zn content were mapped, with their final simultaneous fit providing an R^2^ of 65.4%. All favorable alleles were from AIMP 92901-deriv-08, except for two QTLs on LG4B at position eight and 22 cM. The R^2^ values for the individual putative QTLs for this trait ranged from 9.4 to 30.4% (Table 4). Similarly, their additive effects ranged from 0.6 to 6.7. Using single-environment data, one putative digenic interaction was observed for Zn. 

**Figure 3:**
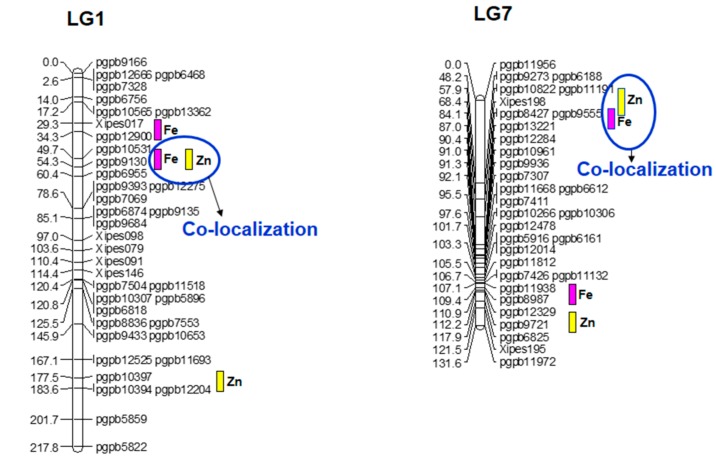
QTL positions for grain Fe and Zn content in (ICMS 8511-deriv × AIMP 92901-deriv-08)-based RIL population. Note that all QTLs are from high grain Fe-Zn content parent AIMP 92901.

Pleiotropism and linkage in quantitative traits are responsible for the correlations among QTLs and, consequently, the detection of co-localized QTLs. Similar to Kumar et al. [14], QTLs for Fe and Zn were co-mapped (Figure 3) on LG1 (major QTL) and LG7 (minor QTL), and favorable alleles for these QTLs were contributed by the male parent. The co-mapping of QTLs for minerals content has been earlier observed in cereals and millet in many studies [14]. Such outcomes indicate that some QTLs control the expression of multiple traits. The co-localization of QTLs also suggests the presence of QTLs with major effects for positively correlated traits [66]. From the crop improvement point of view, co-localized QTLs are of importance for the simultaneous improvement of Fe and Zn content in the grain of pearl millet. 

## 4. Conclusions

Increasing grain iron and zinc content in pearl millet is an important breeding target for the nutritional security of the poor, especially women and children, in the semi-arid tropics. The development of trait-specific mapping populations is the prerequisite to dissect complex traits like micronutrient content in pearl millet. Therefore, an *Iniadi*-derived immortal bi-parental RIL mapping population with 319 lines was developed, segregating for grain iron and zinc content. The high genetic variation in the studied population for grain Fe and Zn content was exploited to detect QTLs for both minerals. Two co-localized QTLs were detected on LG1 and LG7. After further validation, these QTL may be used in marker-assisted breeding programs for the development of high grain Fe and Zn hybrid parental (A-/B- and R-) lines and in marker-assisted population improvement (MAPI) programs globally.

## Figures and Tables

**Table 1:** Details of the diversity array technology (DArT)- and simple sequence repeats (SSRs)-based genetic map of the pearl millet RIL population based on the cross ICMS 8511-deriv × AIMP92901-deriv-08.

Linkage Group	SSR Marker Loci	Skewed SSR Loci	DArT Marker Loci	Skewed DArT Loci	Total Marker Loci	Total Skewed Marker Loci	Skewed Loci (%)	Total Length (cM)	Average Inter-Marker Distance (cM)
1	5	4	33	23	38	27	71	218	5.9
2	2	2	28	23	30	25	83	114	3.9
3	3	3	19	14	22	17	77	84	4.0
4A	3	3	35	23	38	26	68	178	4.8
4B	0	0	5	2	5	2	40	26	6.6
5	3	0	15	6	18	6	33	166	9.7
6	1	1	12	3	13	4	31	47	3.9
7	2	1	30	24	32	25	78	132	4.3
A	0	0	5	4	5	4	80	16.3	4.1
B	1	1	3	3	4	4	100	18.2	6.1
C	0	0	4	2	4	2	50	23.5	7.8
D	1	0	1	1	2	1	50	25.4	25.4
E	2	2	1	1	3	3	100	14.9	7.5
F	0	0	2	2	2	2	100	4.1	4.1
G	0	0	2	0	2	0	0	22.7	22.7
H	0	0	4	3	4	3	75	33.8	11.3
I	2	2	0	0	2	2	100	21.3	21.3
J	0	0	2	2	2	2	100	4.9	4.9
K	0	0	2	2	2	2	100	3.1	3.1
L	0	0	2	2	2	2	100	1.9	1.9
M	1	0	1	1	2	1	50	12.9	12.9
N	1	0	1	1	2	1	50	19.9	19.9
Total	27 (19) ^#^	19 (14)	207 (177)	142 (118)	234 (196)	161 (132)	69 (68)	1187 (964)	5.0 (4.9)

^#^ The values in parentheses show statistics of the base map from LG1 to LG7. RIL: Recombinant inbred line.

**Table 2:** Descriptive statistics of phenotypic values observed in RILs derived from the cross ICMS 8511B × AIMP 92901-08 at ICRISAT-Patancheru.

	8511 (P1)	AIMP (P2)	RILs		P1 vs. P2	P1 vs. RILs	P2 vs. RILs
Trait	Mean	Mean	Mean	Range	Pr > F	Pr > F	Pr > F
Fe	29.8 ± 3.3	124.1 ± 2.9	54.0 ± 0.3	20.0–131.0	**	**	**
Zn	27.7 ± 2.7	86.3 ± 2.4	43.9 ± 0.3	18.2–109.8	**	**	**

Fe = Self-pollinated grain Fe content (ppm); Zn = Self-pollinated grain Zn content (ppm); ** Significant at 1% level.

**Table 3:** Genotypic variance (σ2g), standard error (SE), and operational heritability (H^2^, broad-sense) for the traits observed in the (ICMS 8511-deriv × AIMP 92901-deriv-08)-derived RIL population at ICRISAT-Patancheru.

Trait	σ^2^g	SE	H^2^
Fe	417.98	35.90	0.88
Zn	197.29	17.46	0.83

**Table 4:** Positions and descriptions of QTLs affecting various traits in the (ICMS 8511-deriv × AIMP 92901-deriv-08)-based RIL population.

Trait	QTL ^1^	Marker Interval	Support Interval	LOD ^2^	R^2^ (%)	Additive Effects ^3^
Fe	1/30	*Xipes017-pgpb12900*	26–36	6.22	9.0	4.0
	1/54	*pgpb10531-pgpb9130*	52–56	25.36	31.9	9.7
	3/20	*Xipes188-pgpb6069*	8–26	6.59	9.5	0.4
	4B/8	*pgpb9502-pgpb6039*	4–12	6.87	10.4	−0.6
	7/16	*pgpb11956-pgpb9273*	0–30	7.25	12.5	−1.9
	7/86	*pgpb8427-pgpb13221*	84–90	8.58	12.2	5.3
	7/108	*pgpb11938-pgpb8987*	106–110	8.83	12.5	4.9
	7/120	*pgpb6825-Xipes195*	118–122	9.70	14.0	0.1
	A/0	*pgpb8445-pgpb11206*	0–2	7.67	12.4	4.0
	D/20	*pgpb10660-pgpb8626*	14–24	7.00	11.6	1.2
	E/14	*pgpb10727-Xipes179*	12–14	9.36	14.3	3.1
Zn	1/54	*pgpb10531-pgpb9130*	52–56	23.93	30.4	6.7
	1/182	*pgpb10397-pgpb10394*	178–186	6.50	9.4	1.7
	4B/8	*pgpb9502-pgpb6039*	4–10	7.33	11.1	−0.6
	4B/22	*pgpb10483-pgpb11463*	14–26	6.68	11.6	−2.2
	5/112	*pgpb13229-pgpb12681*	108–118	8.17	11.6	2.7
	7/82	*Xipes198-pgpb8427*	74–88	7.16	10.2	2.7
	7/112	*pgpb12329-pgpb9721*	110–116	7.58	10.9	2.8
	H/16	*pgpb8779-pgpb12691*	10–22	6.68	11.6	2.1

^1^ Leading number: Linkage group; trailing number: QTL position in cM; ^2^ LOD: logarithm of the odds; ^3^ Positive additive effects indicate that the favorable alleles originated from AIMP 92901.

## Supplementary Materials

The following are available online at http://www.mdpi.com/2073-4425/9/5/248/s1, Table S1: Total mapped genome and linkage group lengths for various pearl millet mapping populations.
