# Supplementary material for: Mapping Grain Iron and Zinc Content Quantitative Trait Loci in an Iniadi-Derived Immortal Population of Pearl Millet
Source: Genes (Basel). 2018 May 11;9(5):248. doi: 10.3390/genes9050248 (PMC5977188; doi:10.3390/genes9050248)
Supplement: Supplementary file 1 [file genes-09-00248-s001.pdf]

Supplementary Table 1. Total mapped genome and linkage group lengths for various pearl millet mapping populations

Mapping populations[#]	1[@]	2	3	4	5	6	7	8	9	10
LG \ Pedigree	LGD1-B-10 x ICMP 85410	81B-P6 x ICMP 451-P8	IP18293 x Tift 238D1	W504-1-1x P310-17-B	PT 732B-P2 x P1449-2-P1	ICMB 841-P3 x ICMB 863-P2	ICMB 89111-P6 x ICMB 90111-P6	ICMB 841-P3 x ICMB 863-P2	H77/833-2 x PRLT 2/89-33	ICMB 841-P3 x ICMB 863-P2
1	33.4	77.3	58.9	113.8	172.6	104.9	139.6	116	215.9	374.8
2	36.2	175.8	136.4	31.6	87.9	179	192.3	81.4	370.3	264.3
3	38.3	52.2	62.9	24.2	27.6	15.4	30.2	24.4	77.8	212.6
4	63.2	132.4	110.9	116.6	100	64.3	98.3	54.6	156.1	192.8
5	30.9	102.8	51.9	35.5	30.2	36.9	50.1	62.3	112.0	192.0
6	32.5	58.3	67.8	57.6	83.1	113.1	42.2	147.6	81.7	18.8(A)<br>94.2(B)<br>104.8 (C)
7	13.3	96.9	24.7	39.7	37.6	113.8	195.2	187.2	134.4	294.4
Genome length	287.7	695.7	513.5	421	539	617.4	747.9	673.5	1148	1748.7
Marker size	181	123	33	38	58	91	46	112	321	305
Inter-marker distance	1.59	5.66	15.56	11.08	9.29	6.78	16.26	6.01	3.58	5.73
Population size	133	184	142	175	136	147	172	149	140	106
Reference	Liu et al, 1994	Devos et al, 2000	Azhaguvel et al, 2001	Kolesnikova 2001	Nepolean, 2002	Yadav et al, 2004	Gulia, 2004	Senthilvel et al, 2008	Supriya et al, 2011	Kumar et al, 2016

[#] = Mapping populations from 1 to 8 F2 and from 9 to 10 RILs.
[@] = Mapping distance function for population 1 was Kosambi function.
[$] = Statistics of base map from LG1 to LG7
